# Dysnatremia in Gastrointestinal Disorders

**DOI:** 10.3389/fmed.2022.892265

**Published:** 2022-05-13

**Authors:** Catherine Do, Gretta J. Evans, Joshua DeAguero, G. Patricia Escobar, Henry C. Lin, Brent Wagner

**Affiliations:** ^1^Division of Nephrology, Department of Medicine, Kidney Institute of New Mexico, University of New Mexico Health Science Center, Albuquerque, NM, United States; ^2^New Mexico Veterans Administration Health Care System, Albuquerque, NM, United States; ^3^University of New Mexico Health Sciences Center, Albuquerque, NM, United States

**Keywords:** sodium, homeostasis, hyponatremia, hypernatremia, diarrhea, emesis, chloride

## Abstract

The primary solute of the *milieu intérieur* is sodium and accompanying anions. The solvent is water. The kidneys acutely regulate homeostasis in filtration, secretion, and resorption of electrolytes, non-electrolytes, and minerals while balancing water retention and clearance. The gastrointestinal absorptive and secretory functions enable food digestion and water absorption needed to sustain life. Gastrointestinal perturbations including vomiting and diarrhea can lead to significant volume and electrolyte losses, overwhelming the renal homeostatic compensatory mechanisms. Dysnatremia, potassium and acid-base disturbances can result from gastrointestinal pathophysiologic processes. Understanding the renal and gastrointestinal contributions to homeostatis are important for the clinical evaluation of perturbed volume disturbances.

## Introduction

The kidneys tightly regulate the amount of water and electrolytes in the human body to maintain a consistent internal fluid. They must adjust the balance of excreted water and urine electrolytes in stable conditions and when faced with extreme stress brought on by disease. However, large losses of water and electrolytes through the gastrointestinal tract can overwhelm these impressive homeostatic capabilities, producing dysnatremia, hypokalemia and acid-base disturbances. Diarrhea and vomiting are the two most common gastrointestinal disturbances causing these homeostatic perturbations. We delve into the underlying physiological complexities leading to these abnormalities in the following review article.

## Normal Physiology Under Steady State

A firm grasp of normal gastrointestinal and renal physiology is necessary to understand the electrolyte disturbances caused by gastrointestinal disturbances.

### Sodium and Potassium Distribution in the Body

The ionic composition of intracellular and extracellular compartment in the body differ vastly with transmembrane gradients established by active transport. Sodium (Na^+^) is the main cation in the extracellular space, exerting an effect on cell volume by influencing the movement of water. In other words, Na^+^ exerts an osmotic effect when confined to the extracellular space, behaving as an effective osmole. The sodium concentration measured in plasma water (145–155 mmol/L) is slightly higher than plasma sodium concentration [P_Na+_] (137–142 mmol/L) since plasma is composed of both an aqueous phase (water) and a solid phase (proteins and lipids) (1, 2). Sodium and other electrolytes are found in the water fraction of the plasma. Normally, the plasma contains 93% water and 7% solids (proteins and lipids), thus the normal range of the sodium concentration in plasma water is higher than the corresponding normal range of plasma sodium concentration. Modern methods of plasma sodium measurement including ion-selective electrode utilize plasma dilution to indirectly calculate the plasma sodium concentration under the assumption water makes up 93% of plasma under normal conditions (2, 3). Any reduction in the percentage of plasma water such addition of lipids or protein can lead to a falsely low [${\text{P}}_{\text{Na}}^{+}$] measurement, yielding pseudohyponatremia.

Osmolality is the preferred unit to express concentration in biological fluids where solutes are measured per mass in osmoles (Osm) per kilogram (kg) solvent over osmolarity in which solutes are measured as moles (mol) per liter (L) of solvent, a unit measurement based on volume. In the clinical laboratory, osmolality is a measurable variable, whereas plasma osmolarity is often estimated from sodium, urea nitrogen, and glucose (not accounting for a number of osmoles). Therefore, osmolality (expressed as Osm/kg or mol/kg) is preferred over osmolarity (mol/L) when dealing with biological fluids. Addition of solutes to a solvent will decrease its freezing point, a colligative property, making freezing point depression useful in measuring plasma osmolality. Since the plasma solute concentrations are small (mmol and mOsm), there is little difference between plasma osmolarity and osmolality in practice as noted by Edelman et al. (4). Although sodium is the predominant extracellular cation, it is not the sole determinant of plasma osmolality (*P*_*Osm*_) as estimated in the following Equation.


$$\begin{array}{l} P_{Osm}=2\times \left[P_{Na^{+}}\right]+\frac{\left[BUN\right]}{2.8}+\frac{\left[Glc\right]}{18} \end{array}$$

Blood urea nitrogen (BUN) and glucose (Glc) are measured in mg/dL in the above equation and must be converted to mmol/L by the conversion factors 2.8 for the nitrogen content in BUN and 18 for glucose. Normal plasma osmolality ranges 275–295 mOsmoles/kg of water (2). It should be noted that urea can diffuse between the intracellular and extracellular compartments via transmembrane urea transporters to achieve equilibrium and therefore, exerts less (and time-dependent) osmotic pressure across the cell membrane to influence the movement of water. A solute that is confined to a specific space and cannot freely cross a membrane can influence the direction of movement of water into that space, thus exerting a tonicity force. Therefore, an ion such as Na^+^ or a solute such as glucose, when present in high concentration and confined to the extracellular space, can draw water from the intracellular space, exerting a significant tonic force to change the volume of surrounding cells. The tonicity of a solution is related to its effect on the volume of a cell, thus isotonic solutions have minimal impact on the cell volume in the healthy state.

Intracellular sodium concentration averages 12 mmol/L. Potassium (K^+^) is the main cation in the intracellular space with a concentration ranging 140–150 mmol/L whereas normal plasma K^+^ concentration ranges 3.5–5.5 mmol/liter, reflecting 98% intracellular distribution of total body K^+^ (5–7). The asymmetric distribution of Na^+^ and K^+^ is achieved by sodium-potassium ATPase (Na^+^-K^+^ ATPase) which actively transports Na^+^ and K^+^ in opposite directions to achieve transmembrane gradients necessary for cell signaling, energy storage to facilitate solute transportation and to maintain constant intracellular volume (8). The amounts of potassium in the intracellular compartment and sodium in the extracellular compartment are the main determinants of the fraction of body water in each compartment (9). Edelman articulated through his isotype equilibration studies that plasma sodium “is a reflection of the ratio of the sum of exchangeable monovalent cation (Na_E_ + K_E_) to T.B.W.” (where T.B.W. is total body water) and introduced the concept of exchangeable sodium (${\text{Na}}_{\text{E}}^{+}$) and potassium (${\text{K}}_{\text{E}}^{+}$) in his original equation (4, 10). He devised the classical concept that Na^+^ and K^+^ exists in the human body in an exchangeable form (as free ions or bound to proteins in plasma and interstitial fluid) and an unexchangeable form (bound to cartilage, skin and bone) with plasma water sodium determined by the exchangeable form. Burton Rose later simplified the Edelman equation to its modern version shown here (11):


$$\left[P_{Na^{+}}\right]\;=\;\left(\frac{Na_{E}^{+}\;+\;K_{E}^{+}}{TBW}\right)$$

**Figure 1:**
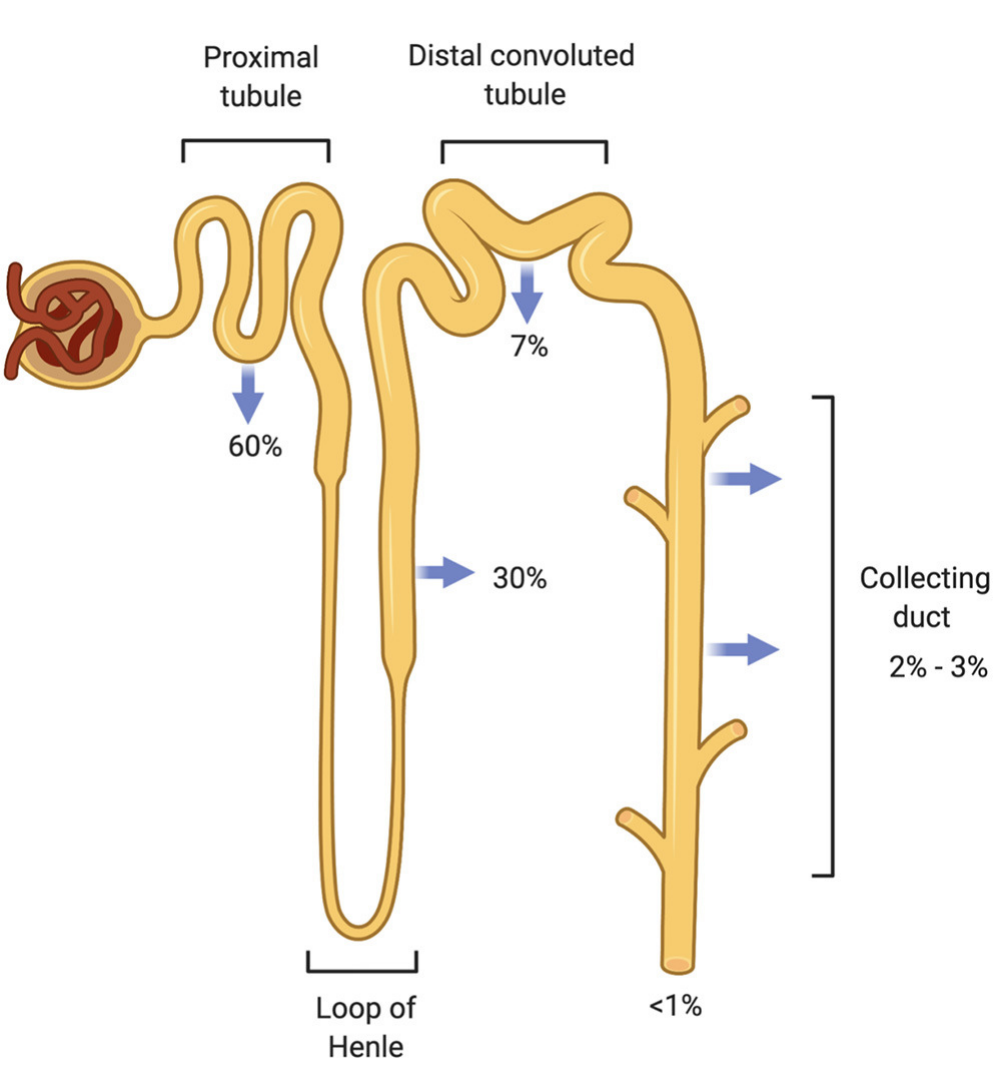
Sodium reabsorption throughout the nephron. The nephron has multiple sodium chloride transporters to reabsorb filtered sodium so that <1% of filtered sodium is excreted from the body. The proximal tubule (PT) reabsorbs the majority of the filtered load (60%) followed by the thick ascending Loop of Henle (TALH) and the distal convoluted tubule (DCT). Very little sodium remains for reabsorption by the time the tubular fluid reaches the cortical collecting duct (CCD). Sodium is conserved through these redundant reabsorption mechanisms.

Nguyen and Kurtz later refined the Rose equation in an attempt to improve its accuracy to predict changes in [${\text{P}}_{\text{Na}}^{+}$], but the simpler Rose formula has proven to be reliable in most clinical cases (12). Daily Na^+^ intake averages ~180 mmol (4.2 g) in men and 150 mmol (3.5 g) in women (13). The kidneys filter plasma at a high glomerular filtration rate (GFR) needed to remove waste products and reabsorb solutes (14). They play an important role in sodium homeostasis where renal sodium excretion occurs to maintain normal extracellular fluid volume and control arterial blood pressure. More than 500 g Na^+^ is extracted from the plasma ultrafiltrate daily in order to excrete the ingested 3 g in urine, demonstrating highly efficient Na^+^ reabsorption capabilities along the nephron to conserve total body Na^+^ (13). Multiple Na^+^ transporters located on the apical renal tubular cell surface facilitate sodium reabsorption throughout the nephron beginning with the Na^+^-H^+^ exchanger in the proximal tubule followed by the Na-K-2Cl transporter in the loop of Henle and the Na-Cl cotransporter in the distal convoluted tubule, and finally the epithelial sodium channel (eNaC) in the collecting duct (Figure 1). Na^+^-K^+^ ATPase, located on the basolateral surface of these tubular cells, serves as the efflux mechanism for sodium to return to the vascular compartment in exchange for entry of blood potassium through the same cells for secretion into the urine. Review of each sodium transporter is outside the scope of this article, so the reader is referred to the Palmer and Schnermann review article for further details (13).

### Antidiuretic Hormone (ADH)

**Figure 2:**
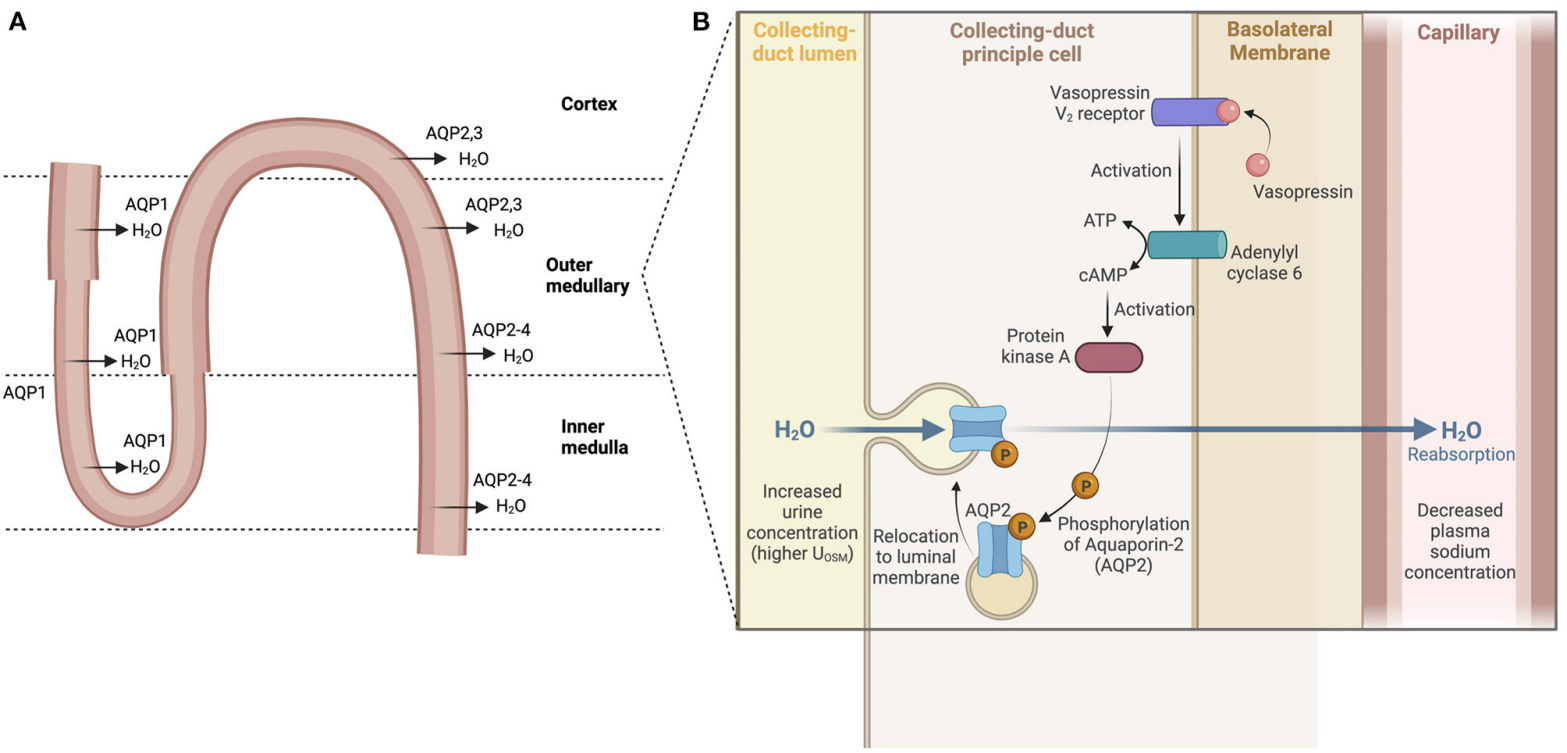
Water reabsorption in the nephron. **(A)** Water is absorbed throughout the nephron via aquaporins (water channels) starting in the proximal tubule and descending loop of Henle. Water reabsorption in the cortical collecting duct reflects the main site of action of ADH. **(B)** ADH binds to vasopressin V2 receptors, activating an adenyl cyclase to produce cAMP which then activates protein kinase A (PKA). Phosphorylation of PKA leads then to the translocation of the water channel protein aquaporin 2 (AQP2) from their cytosolic location to the apical plasma membrane resulting in the movement of water from tubular lumen to blood.

High renal sodium avidity conserves total body sodium. This property of the kidneys plus renal water loss or water conservation in the steady state are key determinants of [${\text{P}}_{\text{Na}}^{+}$] Antidiuretic hormone (ADH) or arginine vasopressin secreted by the posterior pituitary reduces water loss in the urine by promoting renal water reabsorption. ADH binds to vasopressin V2 receptors on the renal collecting duct to activate an adenyl cyclase to produce cAMP which, in turn, activates protein kinase A (PKA). Phosphorylation of PKA leads to the translocation of the water channel aquaporin 2 (AQP2) from its location in cytosolic vesicles to the apical plasma membrane of collecting duct cells resulting in the movement of water from tubular lumen to blood (15, 16) (Figure 2). ADH expression and release are driven by both osmotic and non-osmotic mechanisms. Specifically, rising osmolality detected by an osmoreceptor in the anterior hypothalamus leads to the release of ADH to maintain plasma osmolality between 280 and 295 mOsm/kg (17). ADH release also occurs when arterial baroreceptors detect depletion of circulatory volume (18). Rising plasma osmolality activates osmoreceptors, triggering thirst to increase water intake along with release of ADH (19). Even as ADH both modulates water balance and acts as a vasoconstrictor on smooth muscles of blood vessels, a reflex buffering mechanism exists to prevent a significant rise in arterial blood pressure until the maximal antidiuretic dose of ADH is released. This is the result of a baroreceptor-mediated tonic inhibition of the synthesis and secretion of ADH via vagal afferent nerve input to the pituitary (20). Any disease process that reduces baroreceptor sensitivity would lead to dis-inhibition of synthesis and secretion of ADH or greater release of ADH.

Non-osmotic stimulants of ADH secretion include decreased blood pressure and volume which are sensed by baroreceptors in heart and large arteries. Changes in blood pressure and volume are not as potent stimulators of ADH release as osmotic changes but are powerful in extreme conditions. Nausea and vomiting are also potent non-osmotic stimulators of ADH release (21, 22).

Plasma sodium concentration and ultimately plasma tonicity are closely controlled by water homeostasis mediated by thirst, release of ADH and its effects on renal water reclamation (23).

### Electrolyte and Water Absorption in the Gastrointestinal Tract

**Figure 3:**
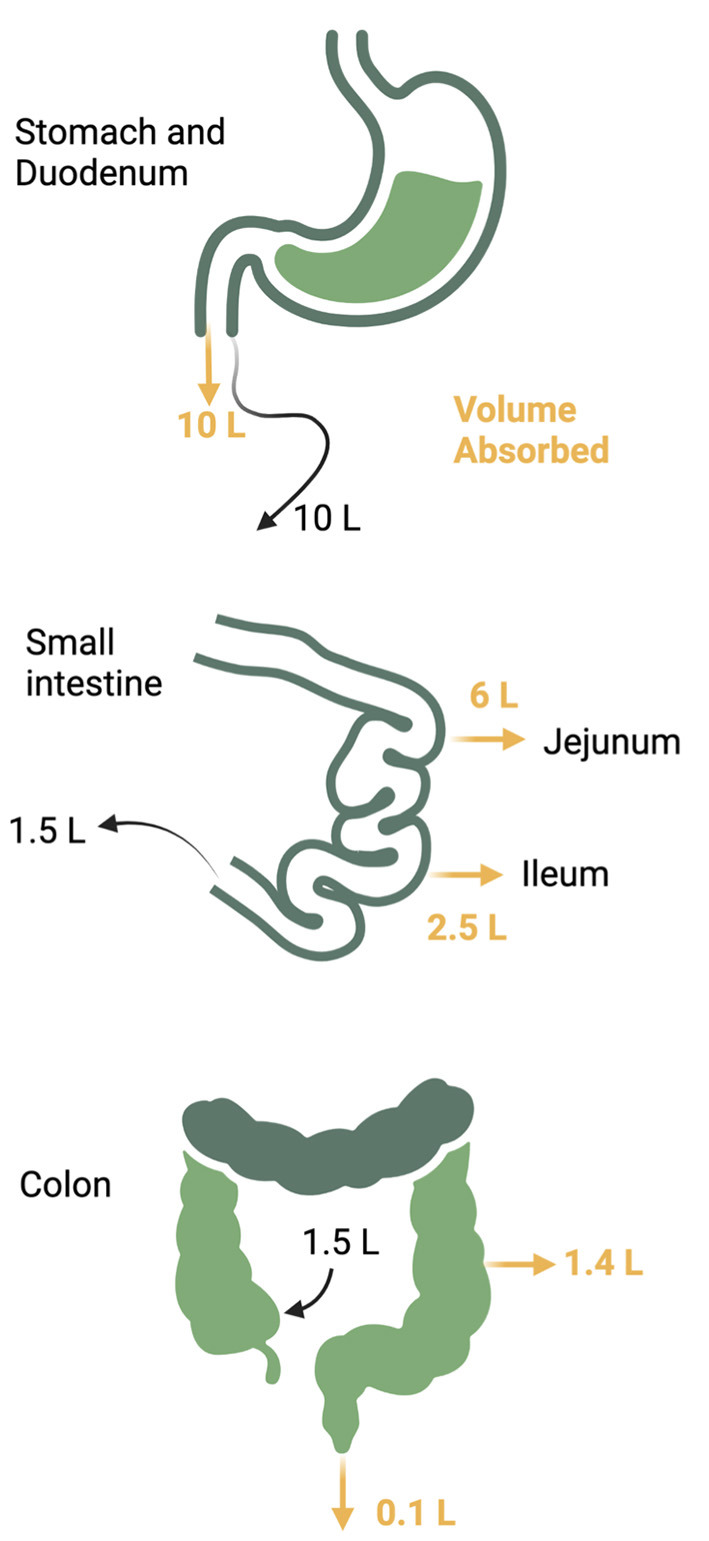
Daily gastrointestinal fluid volume. Up to 10 liters of fluid traverses the gastrointestinal tract daily from both oral take and pancreatic and luminal secretions. The majority of the volume is reabsorbed in the jejunum and ileum with another 1.4 liters reabsorbed from the colon to reduce the stool volume to 0.1 liter per day.

The gastrointestinal (GI) tract absorbs nutrients and fluids daily from dietary intake in addition to its endocrine and immune functions. Daily fluid intake ranges 1.5–2 liters, but secretion of fluid from saliva, gastric juices, pancreatic juices, and bile add more volume to total a daily flux of 10 liters through the upper small intestine (24–26). Fluid, electrolytes, and nutrients are absorbed so that the volume is reduced to 1.5 liters when the gastrointestinal fluids reach the ileocecal valve. The colon absorbs another 1.4 liters so that final stool volume is 0.1 liters (Figure 3). Sodium and water absorption are enhanced in the upper small bowel with the electrolyte content of the intestinal lumen mirroring plasma electrolyte concentrations. The adult gut can absorb up to 1,000 mmol of sodium per day with most of the absorption occurring in the small intestine. The colon absorbs another 50 mmol of sodium and chloride and secretes a small amount of potassium (25). Failure of small intestinal absorption will lead to delivery of large volumes to the colon, overwhelming the limited absorptive capacity of this segment (25). Table 1 summarizes the electrolyte content of GI secretions and fluids.

**Table 1:** Approximate electrolyte content of gastrointestinal secretions*.

**Secretion**	**Sodium [Na^**+**^] mmol/l**	**Potassium [K^**+**^] mmol/l**	**Chloride [Cl^**−**^] mmol/l**	**Bicarbonate [${\text{HCO}}_{3}^{-}$] mmol/l**
Saliva	44	20	-	-
Gastric	70–120	10	100	-
Bile	140	5	100	40–60
Pancreas	140	5	75	70–120
Small intestine	110–120	5–10	105	30
Colon/fecal water	<30	55–75	<30	30

* *Values are from Sobotka et al. (24) and Gennari and Weise (27)*.

**Figure 4:**
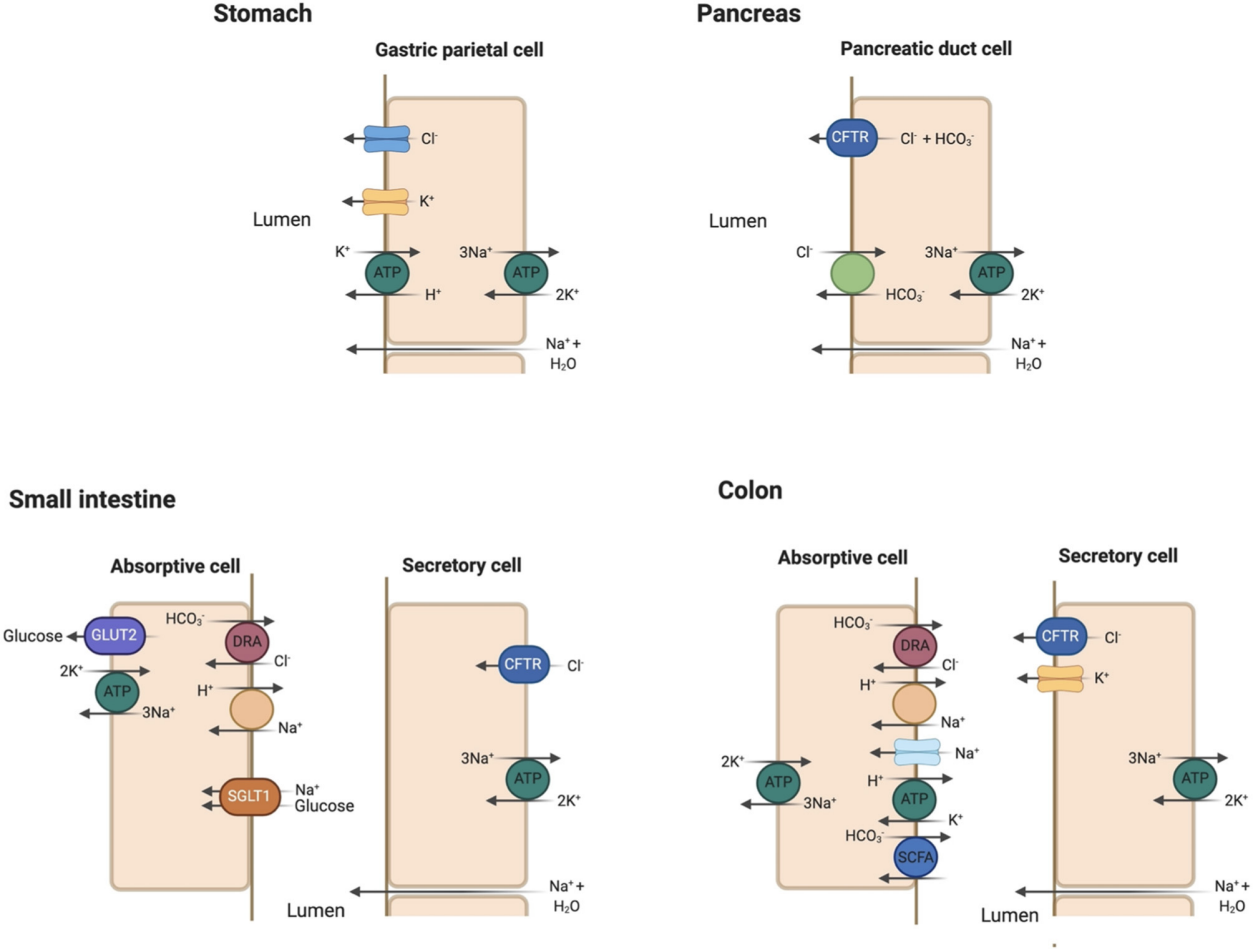
Key apical membrane ion transporters and channels in the gastrointestinal tract. *Stomach:* Luminal Cl^−^ channel and H^+^/K+-ATPase play prominent roles in HCl secretion in the gastric parietal cell. Sodium and water enter the gastric lumen through paracellular mechanisms. *Pancreas:* the Cl^−^/${\text{HCO}}_{3}^{-}$ exchanger and cystic fibrosis transmembrane conductance regulator (CFTR) are essential in bicarbonate secretion to neutralize HCl secreted in the stomach. *Small intestine:* Sodium (Na^+^) absorption occurs via the Na^+^/H^+^ exchanger (NHE). Glucose absorption is coupled to Na^+^ absorption through the sodium-glucose cotransporter 1 (SGLT1) and exits the absorptive cell through glucose transporter 2 (GLUT2). Na^+^/K^+^-ATPase serves as efflux mechanism for absorbed Na^+^. Cl^−^ absorption is coupled with ${\text{HCO}}_{3}^{-}$ excretion through the regulator down-regulated in adenoma (DRA) in the jejunum and ileum. Cl^−^ secretion into the lumen occurs through CFTR. *Colon:* similar transporters for Na^+^ and Cl^−^ absorption as in the small intestine with the addition of epithelial Na^+^ channel (eNaC). K^+^ absorption is coupled to H^+^ secretion via H^+^/K^+^-ATPase. K^+^ secretion occurs under the influence of aldosterone in the colon, a characteristic unique to this segment. Bicarbonate (${\text{HCO}}_{3}^{-}$) is secreted in the colon with the absorption of small chain fatty acids (SCFA).

The stomach is mostly impermeable with negligible absorption. Duodenal mucosa is freely permeable with considerable movement of water and ions in response to osmotic and concentration gradients. The gut contents become isotonic with plasma in the duodenum through bulk water and solute absorption regardless of their original composition in the stomach. Acid is neutralized by bicarbonate (${\text{HCO}}_{3}^{-}$) addition from pancreatic and biliary secretions. Pancreatic secretion of ${\text{HCO}}_{3}^{-}$occurs primarily through the chloride/bicarbonate (Cl^−^/${\text{HCO}}_{3}^{-}$) ion exchanger with [${\text{HCO}}_{3}^{-}$] ranging 70–120 mmol/L in the pancreatic fluid (27). The cystic fibrosis transmembrane conductance regulator (CFTR) recycles Cl^−^ across the apical membrane and regulates the activity of the Cl^−^/${\text{HCO}}_{3}^{-}$ exchanger (27) (Figure 4).

The initial theory that the intestinal tract separates its absorptive and secretory functions spatially with absorption occurring in locations superficial to the luminal surface (villous cells) while secretion arises from cells in crypts has been challenged in recent years (28). Molecular localization experiments have shown that the Na^+^/H^+^ exchanger (NHE3) essential for Na^+^ and fluid absorption and CFTR, NKCC1 (Na^+^ and K^+^ coupled Cl^−^ transporter) and NBCe1 (Na^+^ and ${\text{HCO}}_{3}^{-}$ cotransporter) involved in stimulated Cl^−^ and HCO3^−^ secretion are present together in all upper crypt and villous enterocytes (29). Both the jejunum and ileum absorb and secrete fluid, but absorption normally predominates to reduce the total gut fluid volume to ~1 l/day by the time it reaches the colon. Sodium, chloride and bicarbonate make up the ionic content in the jejunum and ileum with Na^+^ absorption coupled to glucose absorption through bulk flow along osmotic gradients (30). Fordtran and Carter observed in their early studies increased Na^+^ absorption when solutions containing glucose or galactose were infused into the jejunum (30). These findings suggested that Na^+^ absorption is coupled to glucose absorption through an active transport mechanism later identified as the Na^+^/glucose co-transporter SGLT1 (31). Chloride absorption appears to follow that of Na^+^ passively in the jejunum (25) and can be absorbed actively against an osmotic gradient in jejunum, ileum and colon. The sodium-proton (Na^+^/H^+^) exchanger takes up Na^+^ and secretes H^+^ into the intestinal lumen in addition to coupled Na^+^ absorption through SGLT1. Chloride absorption is coupled with ${\text{HCO}}_{3}^{-}$ secretion through the regulator known as down-regulated in adenoma (DRA) encoded by the gene *SLC26A3* (25, 27, 32) (Figure 4). Chloride and bicarbonate exchange predominates by the end of the ileum producing an alkaline solution. Chloride is also secreted into the intestinal lumen as part of Cl^−^ recycling by CFTR located in the intestinal crypts (33, 34). The presence of luminal Cl^−^ affects ${\text{HCO}}_{3}^{-}$ transport in the ileum and colon such as when luminal Cl^−^ is absent, ${\text{HCO}}_{3}^{-}$ is absorbed as a paired ion with Na^+^ (35). When Cl^−^ is present, ${\text{HCO}}_{3}^{-}$ is secreted into the lumen of both these intestinal segments (35). Small intestinal secretory cells lack apical K^+^ channels so that K^+^ absorption occurs through passive diffusion and solvent drag (27).

Water absorption is postulated to be coupled with Na^+^ and glucose transport in the small intestine via SGLT1 and passively in the colon (36, 37). The role of aquaporin channels in water absorption remains unknown despite the expression of multiple aquaporin isotypes throughout the gastrointestinal system including small intestine (38, 39).

Sodium absorption in the colon occurs via eNaC regulated by aldosterone. Bicarbonate secreted into the colon via the Cl^−^/${\text{HCO}}_{3}^{-}$ exchanger is consumed in buffering organic acids produced by colonic bacteria (27). Some of the organic anions produced by this neutralizing reaction are absorbed via a linked ${\text{HCO}}_{3}^{-}$ exchange transporter, but the remainder are excreted in the stool, making up to 30–40 mmol/day of potential alkali lost (27). The colon absorbs K^+^ while secreting H^+^ into its lumen through the apical membrane H^+^/K^+^-ATPase. However, the colon secretes K^+^ more than it absorbs via luminal K^+^ channels which respond to aldosterone by increasing luminal K^+^ content up to 75 mmol/L (27, 39). Aldosterone does not seem to affect K^+^ secretion in other segments such as the ileum, making the colon unique in its response (40). Final stool water volume tends to be small, making net stool K^+^ loss 10–15 mmol/day (27). The major ion transporters important in absorption and secretion are summarized in Table 2.

**Table 2:** Major ion transporters in the small intestine and colon*.

**Transport function**	**Transporter**	**Ion affected**	**Location**
Absorption	Na^+^/H^+^ Exchanger	Na^+^ absorption	Apical membrane of small intestine and colon
	SGLT1	Na^+^, glucose and water	Apical membrane of small intestine
	Epithelial Na^+^ Channel (ENaC)	Na^+^	Apical membrane of distal colon
	DRA	Cl^−^ absorption, ${\text{HCO}}_{3}^{-}$ secretion	Apical membrane of small intestine and colon
Secretion	NKCC1	Na^+^ and Cl^−^ basolateral cell entry for Cl^−^ secretion	Basolateral cell surface of small intestine and colon
	CFTR	Cl^−^ secretion	Apical membrane of small intestine and colon
Absorption and secretion	Na^+^/K^+^ ATPase	Na^+^ cellular extrusion, K^+^ cell entry	Basolateral membrane of epithelial cells in small intestine and colon

* *SGLT1, sodium glucose transporter 1; DRA, downregulated in adenoma; NKCC1, sodium-potassium- 2 chloride contransporter 1; CFTR, cystic fibrosis transmembrane conductance regulator (32)*.

## Electrolyte Disorders in Vomiting

Vomiting can lead to significant volume and electrolyte loss leading to dysnatremia, metabolic alkalosis, and hypokalemia when large volumes are lost. Gastric fluid normally contains 120-160 mmol/L of Cl^−^ balanced by Na^+^, K^+^, and H^+^ with K^+^ concentration up to 10 mmol/L, so vomiting with small volumes losses rarely lead to significant loss of total body electrolyte content or volume. Volume, electrolyte, and acid-base disturbances present in cases of protracted vomiting or nasogastric suction with large volume loss.

Gastric Na^+^ content varies based on the acidity of the stomach content. Hyponatremia ensues when large amounts of Na^+^ is lost with fluid in the gastric content. Hypotonic hyponatremia is defined as [${\text{P}}_{\text{Na}}^{+}$] <135 mmol/L and should be distinguished from pseudo-hyponatremia observed in paraproteinemia or uncontrolled hyperlipidemia when [${\text{P}}_{\text{Na}}^{+}$] is measured by methods requiring pre-measurement plasma sample dilution (3) or trans-locational hyponatremia as seen in hyperglycemia. Hypotonic hyponatremia reflects an excess of water in relation to Na^+^ (41, 42). Volume depletion can precipitate hypotension, which activates the renin-angiotensin-aldosterone system to increase renal tubular Na^+^ and Cl^−^ reabsorption and activates the release of ADH (9). ADH release leads to increased renal tubular water reabsorption, acting in concert with aldosterone to preserve intravascular volume. If hypotonic fluids are ingested or administered in the presence of increased ADH secretion, more water may be reabsorbed than Na^+^, leading to hyponatremia. Reduced GFR from volume depletion may limit renal water excretion, maintaining hyponatremia through water retention. ADH secretion may occur if hypotension is prolonged, perpetuating hyponatremia despite lacking an osmotic stimulus for its release. Nausea or pain can prolong ADH secretion after resolution of hypovolemia, exacerbating hyponatremia.

Hypernatremia can develop in vomiting when extra-renal water loss exceeds sodium loss in the vomitus causing a net water loss. Hypernatremia is defined as plasma sodium exceeding 145 mmol/L and reflects a hypertonic state (9, 23). A rise in plasma osmolality will trigger thirst prompting water intake and ADH release to increase renal water absorption to correct the hyperosmolality. Hypernatremia develops if water is unavailable, thirst drive is impaired, or if the affected are too young or too old to seek water themselves (9, 23).

Recurrent vomiting with large gastrointestinal fluid loss can lead to gastric alkalosis. The loss of this H^+^-rich gastric fluid leads to increased production of HCl in the parietal cells for secretion into the gastric lumen. Bicarbonate, generated as the conjugate anion from HCl production in the parietal cell, is returned back into circulation, raising plasma ${\text{HCO}}_{3}^{-}$, constituting the generation phase of metabolic alkalosis (43). A sudden rise in plasma ${\text{HCO}}_{3}^{-}$ is followed by increased sodium bicarbonaturia and marked kaliuresis (44). However, ongoing loss of Cl^−^-rich gastric fluid leads to total body Cl^−^ depletion, volume depletion, and reduced GFR. Reduced GFR limits excretion of urinary ${\text{HCO}}_{3}^{-}$ by reducing the filtered load of ${\text{HCO}}_{3}^{-}$, contributing to the maintenance of metabolic alkalosis (45). Volume depletion also activates the renin-angiotensin-aldosterone system which contributes to maintenance of metabolic alkalosis. Angiotensin II promotes renal apical Na^+^/ H^+^ exchange in the proximal tubule and basolateral Na^+^/ ${\text{HCO}}_{3}^{-}$ cotransport back to the vascular space in the proximal tubule. Aldosterone stimulates increased H^+^ secretion from the type A intercalated cell in the distal tubule into the tubular lumen with generation of ${\text{HCO}}_{3}^{-}$ that is extruded back into the blood space in exchange for Cl^−^, maintaining a high plasma ${\text{HCO}}_{3}^{-}$. Chloride depletion reduces the distal delivery of tubular Cl^−^ needed for ${\text{HCO}}_{3}^{-}$ secretion, contributing to the maintenance phase of metabolic alkalosis. A characteristic feature of metabolic alkalosis caused by gastric losses is a urinary Cl^−^ concentration <10 mmol/L (27).

Marked kaliuresis accompanying sodium bicarbonaturia in the generation phase may lead to total body K^+^ loss since urinary K^+^ loss may exceed gastric K^+^ loss (44, 46). Secondary hyperaldosteronism increases Na^+^ reabsorption via eNaC in the principal cell, creating an electronegative gradient favorable for K^+^ secretion via the renal outer medullary potassium channel (ROMK), contributing to ongoing hypokalemia. Hypokalemia in turn increases the activity of the H^+^/K^+^-ATPase exchange pumps in the luminal membrane of type A intercalated cells which reabsorb K^+^ in exchange for H^+^ secretion, maintaining metabolic alkalosis. Hypokalemia also reduces the activity of pendrin, the Cl^−^/${\text{HCO}}_{3}^{-}$ exchanger located in type B intercalated cells, contributing to maintenance of metabolic alkalosis (47). Ultimately, hypokalemia and aldosterone increase activity of H^+^/K^+^-ATPase and H^+^-ATPase, respectively, to enhance distal hydrogen secretion while limiting ${\text{HCO}}_{3}^{-}$ excretion to maintain metabolic alkalosis.

### Treatment

Addressing the underlying cause of vomiting is the first step to correct the various electrolyte and acid-base disturbances induced by vomiting. Correction of volume depletion restores intravascular volume, improves GFR and delivers more filtered ${\text{HCO}}_{3}^{-}$ for tubular excretion. In addition, restoration of effective arterial blood volume stops renin-angiotensin-aldosterone and ADH activation, helping to correct hyponatremia. Fluid resuscitation with a chloride-rich crystalloid also corrects Cl^−^ depletion and increases the delivery of urinary Cl^−^ needed for Cl^−^/${\text{HCO}}_{3}^{-}$ exchange via pendrin to effectively excrete ${\text{HCO}}_{3}^{-}$ and correct metabolic alkalosis. Repletion of potassium also helps to reverse metabolic alkalosis.

## Dysnatremia in Diarrhea

The definition of diarrhea is nebulous as most people refer to increased bowel movements or loose or watery stool consistency (26). Researchers have used a stool weight >200 grams daily in a Western diet to define diarrhea (26). Stool weight can increase in those who eat a high fiber diet but is not considered diarrhea since the stool is formed (26). The solute or water loss in the fecal matter better reflects the meaning of the term diarrhea with two broad categories: osmotic and secretory diarrhea. The stool composition of each type of diarrhea discussed below are summarized in Table 3.

**Table 3:** Stool electrolyte composition in various types of diarrhea*.

**Type of diarrhea**	**Sodium (mmol/l)**	**Chloride (mmol/l)**	**Potassium (mmol/l)**	**Bicarbonate (mmol/l)**	**Osmolality (mmol/l)**
PEG	10–20	2–8	9–17	3–7	350–360
Lactulose	33–36	8–10	14–30	2–4	385
Cholera (adult)	130	100	20	44	
Cholera (child)	100	90	33	30	
Congenital chloride losing	>100	>90	>10		

* *PEG, percutaneous endoscopic gastrostomy; Values obtained from Sack et al. (48), Harris et al. (49), Hammer et al. (50) and Abdullah et al. (51)*.

### Osmotic Diarrhea

Osmotic diarrhea occurs when poorly absorbable, low-molecular weight solutes ingested pull water and ions into the intestinal lumen, leading to loose or unformed stools (33). Solutes such as lactulose, mannitol, sorbitol, polyethylene glycol (PEG), magnesium-based antacids or laxatives or lactose in those lactose-intolerant can lead to osmotic diarrhea. Osmotic diarrhea stops once non-absorbable solute has been purged and decreases with fasting. Hammer et al. induced osmotic diarrhea with PEG and lactulose to uncover its pathophysiology. Polyethylene glycol, non-absorbable and not metabolized by colonic bacteria, was administered at increasing doses and served as a contrast to lactulose, which is metabolized by colonic bacteria, to illustrate the differences in stool composition. Increasing osmotic loads of PEG caused a near linear increase in stool water output, averaging 75–80% (50). Stool osmolality was 60–70 mOsmol/kg higher than normal plasma osmolality of 290 mOsmol/kg (50). Stool Na^+^, Cl^−^, and ${\text{HCO}}_{3}^{-}$ concentrations were lower than their respective concentrations in the plasma. PEG-induced diarrhea was associated with very small enteric losses of Na^+^, K^+^, and Cl^−^ (50). Daily fecal losses of Na^+^ ranged from 4–31 mmol/day while fecal K^+^ loss ranged from 6–13 mmol/day and fecal Cl^−^ loss ranged from 1–10 mmol/day with increasing PEG dose (50).

Lactulose was also administered in increasing doses with increased stool weight as observed with PEG, but the stool percent water content rose to an average of 90%, reaching a maximum of 1,100 g/day (50). Organic acid concentration decreased with mean stool osmolality ~90 mosmol/kg higher than plasma osmolality due to colonic absorption of these organic acids. Fecal carbohydrate concentration rose due to bacterial metabolism of lactulose, contributing more of an osmotic driving force for diarrhea (50). Fecal Na^+^ and K^+^ content were higher with higher daily fecal losses compared to PEG-induced diarrhea (50). Shiau et al. observed similar fecal Na^+^ and K^+^ concentrations in patients with lactulose-induced diarrhea (52).

The differences in stool solute composition, fecal water content and stool osmolality between these two types of osmotic diarrhea illustrates how stool losses can determine plasma electrolyte and acid-base disturbances. Some ${\text{HCO}}_{3}^{-}$ is secreted to neutralize these organic acids, so the loss of fecal organic acids is a loss of a potential bicarbonate pool, leading to metabolic acidosis. The greater loss of organic acids in lactulose-induced diarrhea leads to greater losses of obligate cations such as Na^+^ and K^+^. Greater loss of fecal K^+^ can precipitate total body K^+^ depletion, leading to hypokalemia. If fecal water loss exceeds Na^+^ loss, volume depletion and hypernatremia may ensue despite compensatory renal water reabsorption. Hyponatremia occurs if Na^+^ loss exceeds water loss.

### Secretory Diarrhea – Cholera

Secretory diarrhea results from overstimulation of the intestinal tract's secretory capacity with a net secretion of anions (Cl^−^ and ${\text{HCO}}_{3}^{-}$), net secretion of K^+^ or net inhibition of Na^+^ absorption (26). Most cases of secretory diarrhea are due to infection such as cholera. It is characterized by large stool volumes which can exceed 1 l/h, an absence of red or white blood cells in the stool, absence of fever or other systemic symptoms except those related to volume depletion, persistence of diarrhea with fasting, and lack of excess stool osmotic gap (33). Stool osmotic gap (OG) is calculated by the equation OG = 290 – 2([Na^+^] + [K^+^]) where 290 is the assumed plasma osmolality. Unmeasured cations such as magnesium (Mg^2+^), calcium (Ca^2+^), ammonium (NH${}_{4}^{+}$) and organic cations make up the gap with a value >50 considered abnormal (33).

**Figure 5:**
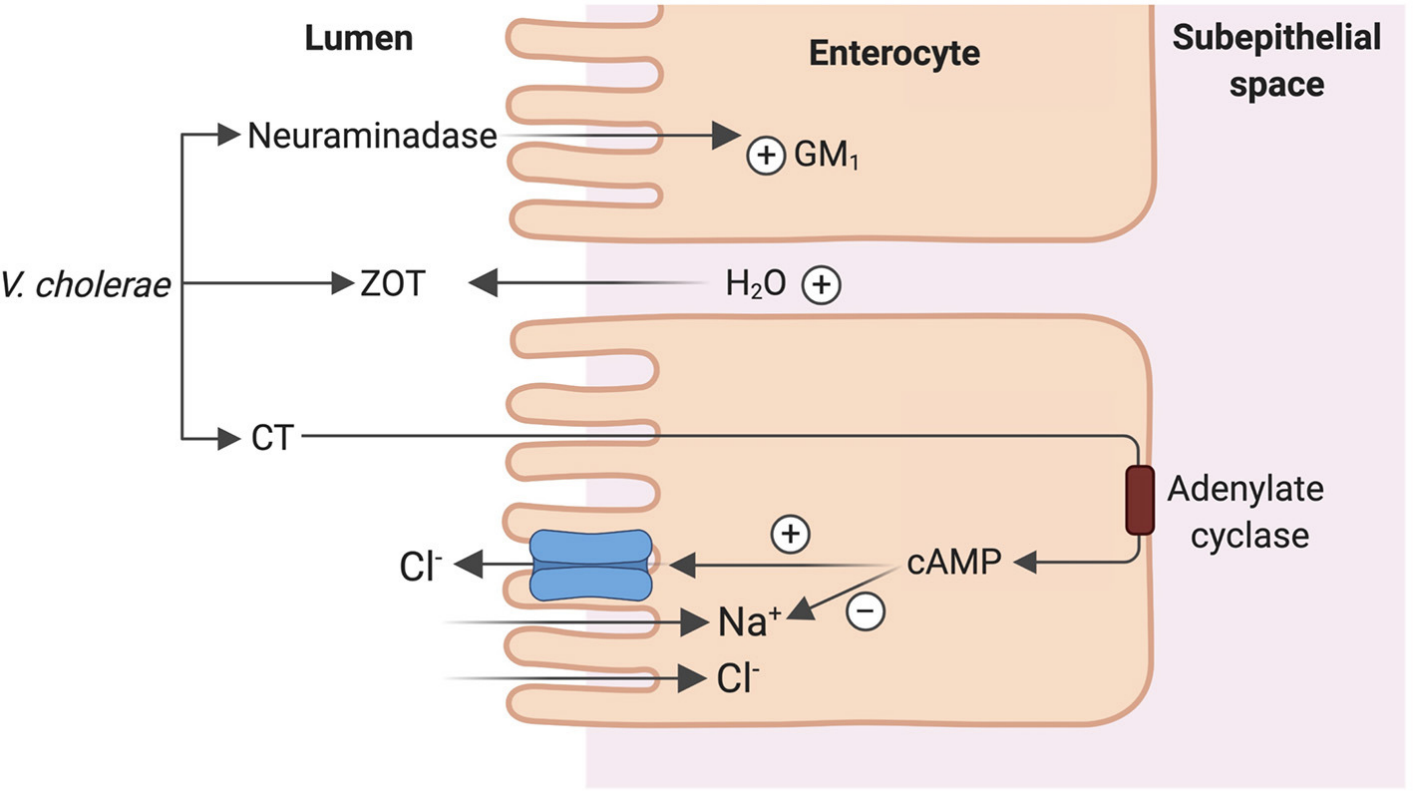
Mechanism of cholera enterotoxicity. *Vibrio cholera* produces 3 toxins that lead to the copious “rice water” cholera diarrhea: cholera toxin (CT), zona occludens toxin (ZOT) and a neuraminidase. CT binding followed by endocytosis leads to increase in cAMP production and ultimately activation of CFTR leading to increased intestinal Cl^−^ secretion and diarrhea. ZOT increases paracellular fluid permeability, contributing to increased stool volume. Neuraminidase increases the number of CT binding sites in adjacent intestinal cells, amplifying the enterotoxicity of CT.

*Vibrio cholera*, the putative Gram-negative bacterial species responsible for copious “rice-water” diarrhea produces the cholera toxin (CT), an 84-kDa protein consisting of a dimeric alpha-subunit and five beta-subunits. The larger A1 subunit of the dimeric alpha-subunit contains the toxic activity. Each of the beta-subunits binds tightly to the GM1 ganglioside abundant in enterocytes brush border followed by endocytosis of the A1 subunit (33). The A1 subunit catalyzes ribosylation of the alpha-subunit of a guanine nucleotide stimulatory protein (Gs), activating adenylyl cyclase to increase cAMP production by 100-fold (53). cAMP activates protein kinase A, which then phosphorylates and activates the apical Cl^−^ channel CFTR, leading to increased intestinal Cl^−^ secretion and diarrhea (53). *V. cholera* produces another enterotoxin, the zona occludens toxin (ZOT), which increases paracellular fluid permeability, contributing to the increased stool volume observed in cholera diarrhea (53). *V. cholera* also produces a neuraminidase that can increase the GM_1_ content of adjacent intestinal cells, increasing cholera toxin binding sites (53) (Figure 5). The final result is large volume diarrhea with increased loss of stool Na^+^, Cl^−^, K^+^, and ${\text{HCO}}_{3}^{-}$. Volume depletion quickly occurs with watery diarrhea up to 1 liter/hour with stool Na^+^ concentration of 130 mmol/L and stool Cl^−^ of 100 mmol/L (48, 49, 54).

Hypernatremia, hypokalemia and metabolic acidosis have all been observed in cholera patients with severe volume loss >3 liters stool volume (54). Hyponatremia can occur in children with significant volume loss (55). Hypokalemia occurs due to fecal K^+^ loss with large volume diarrhea leading to total body K^+^ depletion (54, 55). Metabolic acidosis also occurs due to fecal bicarbonate loss and also lactic acidosis from hypotension and poor tissue perfusion (54, 55).

### Congenital Chloride Diarrhea

Congenital chloride diarrhea due to a defect in the apical Cl^−^/${\text{HCO}}_{3}^{-}$ exchanger results in watery diarrhea with high stool Cl^−^ and metabolic alkalosis (33, 56). The disorder is characterized by an autosomal recessive inheritance pattern with a mutation in the *SLC26A3* gene which encodes for an apical Cl^−^/${\text{HCO}}_{3}^{-}$ exchanger. The exchange mechanism in the ileum and colon is reversed, leading to loss of Cl^−^, producing a profuse chloride-rich diarrhea while increasing absorption of ${\text{HCO}}_{3}^{-}$ (56, 57). Coupled Na^+^/H^+^ transport through the Na^+^/H^+^ exchangers NHE2 or NHE3 leads to intestinal loss of both NaCl and fluid (56). The disease presents early in life with antenatal defects including distended bowel loops and polyhydramnios *in utero* due to fetal secretory “urine like” diarrhea observed on ultrasonography. Premature birth and lack of meconium are other key characteristics of this disease (56). Electrolyte disturbances present immediately after birth manifesting as hypochloremia, hyponatremia, and volume depletion. Stool volumes can range from 2–7 liters daily with lifelong intestinal loss of electrolytes (56). Diagnosis is based on clinical presentation confirmed by high fecal Cl^−^ concentration after repletion of fluid and electrolytes (51, 56). Hypochloremic metabolic alkalosis results from increased intestinal ${\text{HCO}}_{3}^{-}$ absorption (57). Disease management includes salt substitution therapy to prevent Cl^−^ and volume loss.

### Treatment

Treatment of each electrolyte and acid-base disorder accompanying each type of diarrhea includes addressing of the underlying cause appropriately followed by stabilization of hemodynamics, volume repletion and correction of electrolyte disturbances. Aggressive volume repletion either through oral rehydration or intravenous fluids is recommended for cholera patients to make up for the volume already lost and to keep up with ongoing losses. Oral solutions containing both glucose and Na^+^ take advantage of normal coupled intestinal Na^+^/glucose transport to deliver the needed NaCl for volume repletion essential in saving lives. The observation that the addition of glucose to oral salt solutions increases intestinal Na^+^ absorption in cholera treatment shed light on the mechanism of coupled Na^+^/glucose absorption and ultimately led to the discovery of SGLT1 (58).

In those with hyponatremia, slow correction of plasma sodium is recommended to avoid neurologic sequelae from rapid correction. Antibiotic treatment may be indicated for severe infectious diarrhea such as in cholera. Frequent antibiotic use may lead to antimicrobial resistance of *V. cholera*, so its use is reserved for severe cases.

## Conclusion

Both the gastrointestinal and renal systems work in concert to maintain a steady physiological state. The gastrointestinal tract absorbs necessary nutrients and water while the kidneys work to excrete waste products and fine tune the extracellular solute composition. Gastrointestinal losses of sodium, chloride, bicarbonate, potassium and water can overwhelm renal compensatory mechanisms, yielding electrolyte and acid-base disturbances. Understanding of the pathophysiologic mechanisms provides insight into normal physiology and informs the conventional wisdom guiding therapy.

## Author Contributions

CD organized and drafted the final narrative review, selected the figures and commissioned the medical art from a professional artist, constructed the tables, and compiled the majority of the references. GJE, JD, GPE, and HL reviewed the manuscript, figures and tables, and gave input on the final version. BW put together the initial draft of the manuscript along with references, reviewed, and gave editorial input on the final draft. All authors contributed to the article and approved the submitted version.

## Funding

This project was supported in part by the Dedicated Health Research Funds of the University of New Mexico School of Medicine allocated to the Signature Program in Cardiovascular and Metabolic Disease (BW) and the Research Allocation Committee (BW, C-2459-RAC, New Mexico Medical Trust). This project was supported by the National Center for Research Resources and the National Center for Advancing Translational Sciences of the National Institutes of Health through Grant Number UL1TR001449 (BW, CTSC/DCI Kidney Pilot Project CTSC004-12). BW has a user agreement with the Center for Integrated Nanotechnologies (2019AU0120), metabolomics vouchers from the Autophagy Inflammation and Metabolism (AIM) Cener (NIH P20GM121176). UNM Brain & Behavioral Health Institute (BW, Grants 2018-1008 and 2020-21-002), metabolomics voucher from the AIM Center (BW, NIH P20GM121176). Winkler Bacterial Overgrowth Research Fund (HL).

## Dedication

This work was dedicated to CD.

## Conflict of Interest

The authors declare that the research was conducted in the absence of any commercial or financial relationships that could be construed as a potential conflict of interest.

## Publisher's Note

All claims expressed in this article are solely those of the authors and do not necessarily represent those of their affiliated organizations, or those of the publisher, the editors and the reviewers. Any product that may be evaluated in this article, or claim that may be made by its manufacturer, is not guaranteed or endorsed by the publisher.
